# Primary Cilia, Ciliogenesis and the Actin Cytoskeleton: A Little Less Resorption, A Little More Actin Please

**DOI:** 10.3389/fcell.2020.622822

**Published:** 2020-12-17

**Authors:** Claire E. L. Smith, Alice V. R. Lake, Colin A. Johnson

**Affiliations:** Leeds Institute of Medical Research at St. James’s, University of Leeds, Leeds, United Kingdom

**Keywords:** drug screen, ROCK inhibitors, ciliopathies, polycystic kidney disease, ciliogenesis, actin cytoskeleton, cilia, cytoskeletal drugs

## Abstract

Primary cilia are microtubule-based organelles that extend from the apical surface of most mammalian cells, forming when the basal body (derived from the mother centriole) docks at the apical cell membrane. They act as universal cellular “antennae” in vertebrates that receive and integrate mechanical and chemical signals from the extracellular environment, serving diverse roles in chemo-, mechano- and photo-sensation that control developmental signaling, cell polarity and cell proliferation. Mutations in ciliary genes cause a major group of inherited developmental disorders called ciliopathies. There are very few preventative treatments or new therapeutic interventions that modify disease progression or the long-term outlook of patients with these conditions. Recent work has identified at least four distinct but interrelated cellular processes that regulate cilia formation and maintenance, comprising the cell cycle, cellular proteostasis, signaling pathways and structural influences of the actin cytoskeleton. The actin cytoskeleton is composed of microfilaments that are formed from filamentous (F) polymers of globular G-actin subunits. Actin filaments are organized into bundles and networks, and are attached to the cell membrane, by diverse cross-linking proteins. During cell migration, actin filament bundles form either radially at the leading edge or as axial stress fibers. Early studies demonstrated that loss-of-function mutations in ciliopathy genes increased stress fiber formation and impaired ciliogenesis whereas pharmacological inhibition of actin polymerization promoted ciliogenesis. These studies suggest that polymerization of the actin cytoskeleton, F-actin branching and the formation of stress fibers all inhibit primary cilium formation, whereas depolymerization or depletion of actin enhance ciliogenesis. Here, we review the mechanistic basis for these effects on ciliogenesis, which comprise several cellular processes acting in concert at different timescales. Actin polymerization is both a physical barrier to both cilia-targeted vesicle transport and to the membrane remodeling required for ciliogenesis. In contrast, actin may cause cilia loss by localizing disassembly factors at the ciliary base, and F-actin branching may itself activate the YAP/TAZ pathway to promote cilia disassembly. The fundamental role of actin polymerization in the control of ciliogenesis may present potential new targets for disease-modifying therapeutic approaches in treating ciliopathies.

## Introduction

**FIGURE 1:**
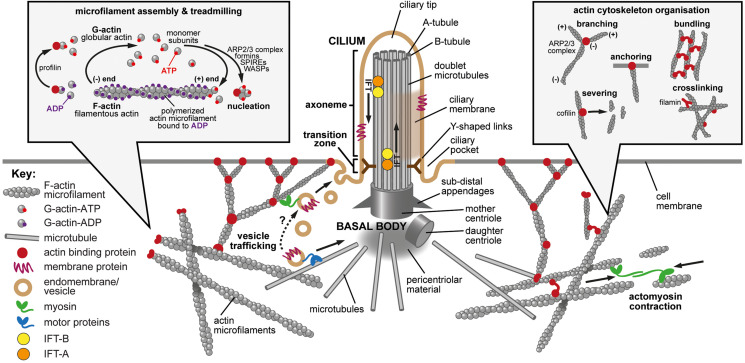
The primary cilium, actin dynamics and the actin cytoskeleton. Main panel: The primary cilium and the actin cytoskeleton. The primary cilium forms when the mother centriole docks at the apical membrane to form the basal body. Microtubules (gray cylinders) then nucleate at the basal body to initiate formation of the axoneme with a 9+0 microtubule arrangement. The sub-distal appendages mediate microtubule anchoring, and the pericentriolar material may act as a microtubule organizing center. Y-shaped links (dark brown) connect the microtubule doublets to the ciliary membrane at the ciliary transition zone, which acts as a gate to control the entry and exit of proteins and lipids. Intraflagellar transport (IFT) protein complexes (orange and yellow) transport cargo (indicated by arrows) along the axoneme by using microtubule based motor proteins. The ciliary pocket is an endocytic membrane domain around the base of the cilium implicated in actin dynamics, the transport of membrane associated proteins to cilia and in ciliary disassembly. The ciliary tip is a source of extracellular vesicles and is involved in early ciliary disassembly through decapitation. The actin cytoskeleton modulates ciliogenesis through effects on both vesicle trafficking (**lower left**; tan symbols and dashed arrow) and actin cytoskeleton remodeling mediated by acto-myosin contractions (**lower right**; green symbols and arrows). Actin-binding proteins are indicated by dark red symbols. **Inset left:** Microfilament assembly and treadmilling. Actin is present in cells as both a monomer (globular, G-actin) and a polymer (filamentous, F-actin). Actin binds and can hydrolyze ATP to ADP. There is preferential additional of ATP-bound monomers to the (+) end of the polymer and ATP is hydrolyzed upon filament assembly. The preferential dissociation of ADP-bound actin results in a “treadmilling” effect, whereby the F-actin filaments exhibit net growth at their (+) ends and net dissociation at their (−) ends. Both polymerization and depolymerization require actin binding proteins. Profilin can sequester G-actin from the pool of actin monomers but can also catalyze the exchange of ADP to ATP, converting monomers to the more polymerizable ATP-bound form. Actin polymerization requires nucleating factors, such as Spire or formins, or the actin-related protein complex (ARP2/3). Actin depolymerization remains incompletely understood but members of the yeast actin depolymerization factor (ADF)/cofilin family can enhance dissociation of monomers from the (−) end. **Inset right:** Actin cytoskeleton organization. Many actin binding proteins can alter the arrangement and structure of F-actin. ARP2/3 can elicit F-actin branching. Members of the yeast actin depolymerization factor (ADF)/cofilin family can both enhance dissociation of G-actin monomers from the (−) end and can sever filaments to produce additional (−) ends. Filamins and various other actin binding proteins can crosslink actin to form complex networks, can anchor F-actin to membranes and can bundle actin into stress fibers.

Primary cilia are microtubule-based organelles that extend from the apical surface of the majority of mammalian cells (Figure 1). Cilia act as “cellular antennae” in vertebrates, receiving diverse inputs from the extracellular environment and transducing signals in response. These signal inputs vary but include mechanical stimuli, for example urine fluid shear stress in kidney epithelial cells (Schwartz et al., 1997); proteins, such as those of the Sonic Hedgehog (Shh) pathway (Huangfu et al., 2003); and low molecular weight chemicals such as dopamine (Atkinson et al., 2015). The specialized cilium of the photoreceptor, the retinal outer segment, is also capable of sensing and transducing signals in response to light (Molday and Moritz, 2015). Cilia responses contribute to the control of developmental signaling and cell proliferation, as well as the establishment of cell polarity (Ross et al., 2005).

Mutations in ciliary genes cause a group of inherited Mendelian developmental disorders termed ciliopathies. Ciliopathies are individually rare but collectively common, affecting up to 1 in 725 people (Wheway et al., 2019). These heterogeneous conditions include kidney diseases, blinding disorders, obesity associated diseases, severe neurodevelopmental abnormalities and skeletal dysplasias [reviewed in Tobin and Beales (2009) and Waters and Beales (2011)]. Ciliopathies can result from defects in the organization or function of the main ciliary components such as the basal body or transition zone, or can be due to deficits in the trafficking of ciliary axoneme components or the transcriptional regulation of ciliogenesis (Reiter and Leroux, 2017).

Treatment options for ciliopathies have been limited to date and have focused on alleviating symptoms rather than treating the causes. Although antisense oligonucleotides, gene replacement therapies and gene editing represent possible future therapeutic avenues for ciliopathies, none are yet licensed (Kim and Kim, 2019). Currently, only one drug is licensed for the treatment of any ciliopathy, Tolvaptan, which has been shown to slow the decline of renal function for patients with autosomal dominant polycystic kidney disease (ADPKD) (Torres et al., 2012, 2017). Tolvaptan is a selective vasopressin V2 receptor antagonist, which ultimately decreases cAMP, reduces activation of protein kinase A and decreases the number of aquaporin channels in the collecting ducts of the kidney (Torres et al., 2012, 2017). This increases water excretion and helps to reduce cyst swelling, slowing decline in kidney function (Gattone et al., 2003; Wang et al., 2008; Meijer et al., 2011; Torres et al., 2012). Unfortunately, Tolvaptan is poorly tolerated due to its common side effects, including polyuria, increased thirst, pollakiuria and xerostomia (Gansevoort et al., 2016), as well as liver damage that resolves upon discontinuation of the drug treatment (Torres et al., 2012). Many other trials are ongoing, including agents to reduce obesity in Bardet-Biedl syndrome and Alström syndrome (NCT03746522) and neurotrophic (NCT00447993 and NCT0447980), anti-apoptotic drugs (Rossetti et al., 1990; Piano et al., 2013; Byrne et al., 2016) and antioxidants (Dias et al., 2018) to treat retinal diseases. Pharmacological agents (for example, the cyclin-dependent kinase inhibitors (R)-roscovitine or the derivative (S)-CR8) that shorten cilia have been tested in animal models of ADPKD with some success (Husson et al., 2016). In addition, many small molecule screens have highlighted several promising compounds and target signaling pathways for further testing (Khan et al., 2016; Booij et al., 2017; Kim et al., 2018).

Understanding of cilia and ciliogenesis will be key to formulating new treatments for ciliopathies. Cilia form cells in G_1_ or G_0_ when the mother centriole docks at the apical cell membrane to form the basal body. This event prompts the local clearance of the layer of cortical actin bound to the plasma membrane (Francis et al., 2011). Microtubules then nucleate at the basal body to initiate the formation of the axoneme. Ciliogenesis is therefore dependent on the cell cycle, is modified by inputs from signaling pathways, but is also heavily influenced by the actin cytoskeleton.

The actin cytoskeleton is composed of microfilaments that are formed from filamentous (F) polymers of globular G-actin subunits (Lodish et al., 2000; Figure 1, left inset). Actin filaments are organized into bundles and networks that are attached to the cell membrane by diverse cross-linking proteins. During cell migration, actin filament bundles form either radially at the leading edge or as axial stress fibers. Early studies demonstrated that loss-of-function mutations in ciliopathy genes increased stress fiber formation and impaired ciliogenesis (Dawe et al., 2007; Valente et al., 2010) whereas pharmacological inhibition of actin polymerization promoted ciliogenesis (Bershteyn et al., 2010; Kim et al., 2010; Sharma et al., 2011). These studies suggested that polymerization of the actin cytoskeleton, F-actin branching and the formation of stress fibers all negatively influence primary cilium formation, whereas depolymerization or depletion of actin enhances ciliogenesis (Avasthi and Marshall, 2012; Malicki and Johnson, 2017). Further support that perturbation of the actin cytoskeleton affects ciliogenesis came from a functional genomics screen to identify positive and negative regulators of ciliogenesis (Kim et al., 2010).

Here we review the current understanding of how the actin cytoskeleton influences ciliogenesis and cilia-dependent cellular functions, such as the maintenance of apico-basal polarity, and the mechanistic basis for these effects. We also discuss the potential for the use of this knowledge to highlight new targets for disease-modifying therapeutic approaches in treating ciliopathies. We do not discuss the roles of signaling pathways mediated by cilia that affect the actin cytoskeleton. Instead, we direct the reader to another recent review which covers this topic (Brücker et al., 2020).

## The Actin Cytoskeleton

Actin was discovered in 1887, initially named “myosin ferment” (Halliburton, 1887), it was then isolated from muscle preparations in 1942 (Straub, 1942). It was not until the 1960s that actin was detected in non-muscle cells (Hatano and Oosawa, 1966; Miki-Noumura and Oosawa, 1969). Actin is an abundant, intracellular cytoskeletal protein that is ubiquitous in eukaryotic cells. There are different isoforms, each encoded by one of six genes in mammals [reviewed in Perrin and Ervasti (2010)]. Actin also undergoes various post translational modifications that are important to its functions [reviewed in Varland et al. (2019)]. It can be present as a free monomer, namely globular actin (G-actin), or polymerized into linear filamentous actin (F-actin) (Straub, 1943). F-actin is a major component of the cytoskeleton, a dynamic network that supports and maintains cellular shape and polarity, whilst also enabling cells to migrate. F-actin is present in cells in part as a microfilament network, which makes up the cortical actin layer that supports the cell membrane. The spontaneous formation of actin filaments in high salt conditions hindered efforts to crystallize actin for structural analysis. High resolution crystal structures of both G-actin and F-actin were finally reported in 1990 (Holmes et al., 1990; Kabsch et al., 1990). The 43kDa G-actin molecule is folded into two similarly sized domains, separated by a cleft that harbors a binding pocket for small molecules, for example ATP *in vivo* or inhibitors (Kabsch et al., 1990).

F-actin is a dynamic polymer with structural polarity due to the common orientation of its subunits. It consists of two twisted helices with an approximate diameter of 5–9 nm (Holmes et al., 1990). Conventional nomenclature for each filament end is derived from the appearance of microfilaments during transmission electron microscopy imaging of samples treated with myosin S1 fragments (the head and neck domains of non-muscle myosin II), subsequently fixed with tannic acid (Begg et al., 1978). Images show myosin-stained actin filaments as “feather-ended arrows.” The positive (+) end is the “feathered” barbed end of the “arrow,” for which myosin molecules are the “feathers” and actin the “arrow” shaft. Conversely, the minus (−) end is named the pointed end because it is not decorated with myosin in this context (Figure 1, left inset).

The majority of studies of actin polymerization and depolymerization have been carried out *in vitro* rather than *in vivo*. *In vivo* dynamic actin processes are estimated to occur at least 100 times faster than *in vitro* (Zigmond, 1993) and are likely controlled by the cumulative effects of many different proteins, meaning that the *in vivo* processes are hard to study. The complexity and speed of signals and responses also show how specific and rapid the modulation of the actin cytoskeleton can be to different stimuli.

Within cells, both polymerization and depolymerization require actin binding proteins (Supplementary Table 1 and Figure 1, right inset). Actin polymerization occurs when G-actin monomers nucleate to initiate the formation of the F-actin polymer. This requires nucleating factors, such as Spire or formins, or the actin-related protein complex (ARP2/3) which can also act to create branches within filaments (Goley and Welch, 2006; Dominguez, 2009). Many other proteins alter actin polymerization and depolymerization kinetics through a variety of other mechanisms. These include binding actin monomers and capping, crosslinking and stabilizing actin filaments (Figure 1, right inset). There are also many actin-binding proteins responsible for actin depolymerization, although this remains incompletely understood. Members of the yeast actin depolymerization factor (ADF)/cofilin family can both enhance dissociation of monomers from the (−) end (Carlier et al., 1997) and can sever filaments to create additional (−) ends (Maciver et al., 1998).

F-actin branching is important in the formation of cellular protrusions, including cilia, lamellipodia and microvilli, and is mediated through the action of the actin-related protein complex (ARP2/3) and Wiskott-Aldrich syndrome proteins (WASp) (Khaitlina, 2014). ARP2/3 creates branches at 70° to the plane of the original actin filaments which help to establish a supportive meshwork for membrane protrusions (Mullins et al., 1998). Branching is an important consideration in disassembly too, since ADF/cofilin preferentially disassembles branched networks rather than either parallel or antiparallel filament bundles (Gressin et al., 2015). Filament severing then occurs at the boundaries between bare and cofilin-bound filament segments, so that cofilin binding density regulates overall filament length (Suarez et al., 2011).

Actin binds and can hydrolyze ATP to ADP (Pollard et al., 2000). This ability, in part, creates the polarity of the actin filaments, along with the preferential additional of monomers to the (+) end of the polymer. Although ATP hydrolysis is not itself required for polymerization (Cooke, 1975), the critical concentration of actin required for polymerization to occur is lower when ATP is bound to G-actin monomers, than when ADP is bound. ATP is hydrolyzed upon filament assembly and makes filaments stiffer (Janmey et al., 1990), but this process lags behind monomer additions, creating a gradient of decreasing ATP-bound actin towards the (−) end of the filament (Carlier et al., 1984; Carlier, 1990). The preferential dissociation of ADP-bound actin results in a “treadmilling” effect, whereby the F-actin filaments exhibit net growth at their (+) ends and net dissociation at their (−) ends (Wegner, 1982). The effect of treadmilling is to create protrusive force in one direction which can be harnessed by motile cells through the formation of structures such as lamellipodia, which are transient actin filament-filled membrane protrusions (Atilgan et al., 2006). Microvilli are also cellular membrane projections formed by treadmilling (Lodish et al., 2000). These dynamic equilibria within actin regulation present another opportunity for modulation by actin binding proteins. Some proteins act by sequestering ATP-bound G-actin monomers to negatively affect filament growth, for example, thymosin beta 4 (Carlier et al., 1993). Others enhance the nucleotide exchange properties of actin to promote growth, for example profilin (Goldschmidt-Clermont et al., 1991).

F-actin also forms microfilament bundles which, together with non-muscle myosin-based motor proteins and actin cross-linking proteins, form stress fibers. Stress fibers are contractile bundles of 10–30 actin filaments, crosslinked by alpha-actinin, found in non-muscle cells with important roles in adhesion, migration, morphogenesis and mechanotransduction (Tojkander et al., 2012). Within stress fibers, myosin II protein can slide along individual actin filaments but non-muscle myosin II can also form bipolar filaments whereby the myosin II heads can also attach to different actin filaments, sliding one actin filament against another to create contractile force (Vicente-Manzanares et al., 2009). Stress fibers vary greatly in their morphology and attachment to focal adhesions, and the processes of their formation and modulation are incompletely understood. Cytokinetic rings, which form during the final step of cell division are another example of a contractile, non-muscle, acto-myosin structure in animal cells (Miller, 2011).

Overall, the highly dynamic and responsive turnover of actin microfilaments enables the rapid reorganization of the actin cytoskeleton in response to extracellular and intracellular cues, particularly for processes dependent on membrane remodeling such as endocytosis and cytokinesis (Fürthauer and González-Gaitán, 2009) and the long-range intracellular transport of vesicles (Schuh, 2011). The large number of proteins that actin is known to interact with (>150 actin binding proteins) provides extensive opportunity for localized fine-tuning of the actin cytoskeleton (Schoenenberger et al., 2011).

## Actin Inhibitors and Detection Reagents

Pharmacological perturbation of actin dynamics has been used to study actin structures within cells. Chemicals that destabilize or stabilize actin filaments, or that prevent or enhance polymerization, have been used extensively. However, results need to be interpreted with caution, since these agents cause global and extreme alterations of the actin cytoskeleton rather than the small, localized changes that would be typical *in vivo*.

The chemicals most often used to destabilize actin experimentally are a group of fungal metabolites called cytochalasins. Cytochalasin D is most often used to perturb actin dynamics because of its favorable inhibition kinetics and better specificity in comparison to other cytochalasins (Cooper, 1987). Cytochalasin D resembles capping proteins which block the extension of actin filaments, since it binds the barbed (+) end of the filament (Cooper, 1987). Cytochalasin D also binds actin monomers and hydrolyses ATP to decrease the pool of ATP-bound actin available for polymerization (Goddette and Frieden, 1986). Treatment of the ciliated hTERT RPE-1 cell-line, at low density in growth-promoting medium, with 0.5 μM cytochalasin D significantly induced ciliogenesis within 8–12 h (Kim et al., 2010; Nagai and Mizuno, 2017). Cytochalasin D treatment at 200 nM was also shown to increase cilia length after only 1 h, in parallel with actin cytoskeleton remodeling that promoted the directional trafficking of ciliary vesicles towards the ciliary base (Kim et al., 2015). Additionally, the authors showed that cytochalasin D treatment resulted in inactivation of the Hippo pathway transcriptional co-activators YAP (yes-associated protein) and TAZ (transcriptional coactivator with PDZ-binding motif, also known as WWTR1) (Kim et al., 2015). These act as the two primary sensors of a cell’s structure, shape and polarity (Piccolo et al., 2014) and typically promote cell proliferation when active (Pan, 2010).

Latrunculin was originally isolated from various marine sponges and is also used experimentally to depolymerize actin (Spector et al., 1983). Latrunculin A is the more potent of the two most common isoforms (A and B) (Spector et al., 1989). Latrunculin binds actin monomers to prevent them from polymerizing (Coué et al., 1987; Morton et al., 2000). Treatment of hTERT RPE-1 cells with 1 μM latrunculin B significantly induced ciliogenesis within 24 h (Nagai and Mizuno, 2017). Jasplakinolide is another compound originally isolated from marine sponges that both promotes actin polymerization and stabilizes filaments (Holzinger, 2010). It is cell membrane permeable, making it ideal for the treatment of live cells (Matthews et al., 1997), and treatment of hTERT RPE-1 cells with 1 μM jasplakinolide significantly induced ciliogenesis within 12 h (Nagai and Mizuno, 2017). However, jasplakinolide treatment did not increase in ciliogenesis in cells plated at higher density (Nagai and Mizuno, 2017), contradicting the predicted effect of a chemical that promotes actin polymerization and should therefore suppress ciliogenesis. However, similar to the effect of the actin destabilizer cytochalasin D (Kim et al., 2015), jasplakinolide treatment also caused cell rounding, reduced cell adherence and proliferation, and inactivation of the transcriptional co-activator YAP (Nagai and Mizuno, 2017). This reiterates the point that pharmacological perturbation of the actin cytoskeleton can cause unexpected effects on ciliogenesis, likely due to the off-target effects of the inhibitor.

**FIGURE 2:**
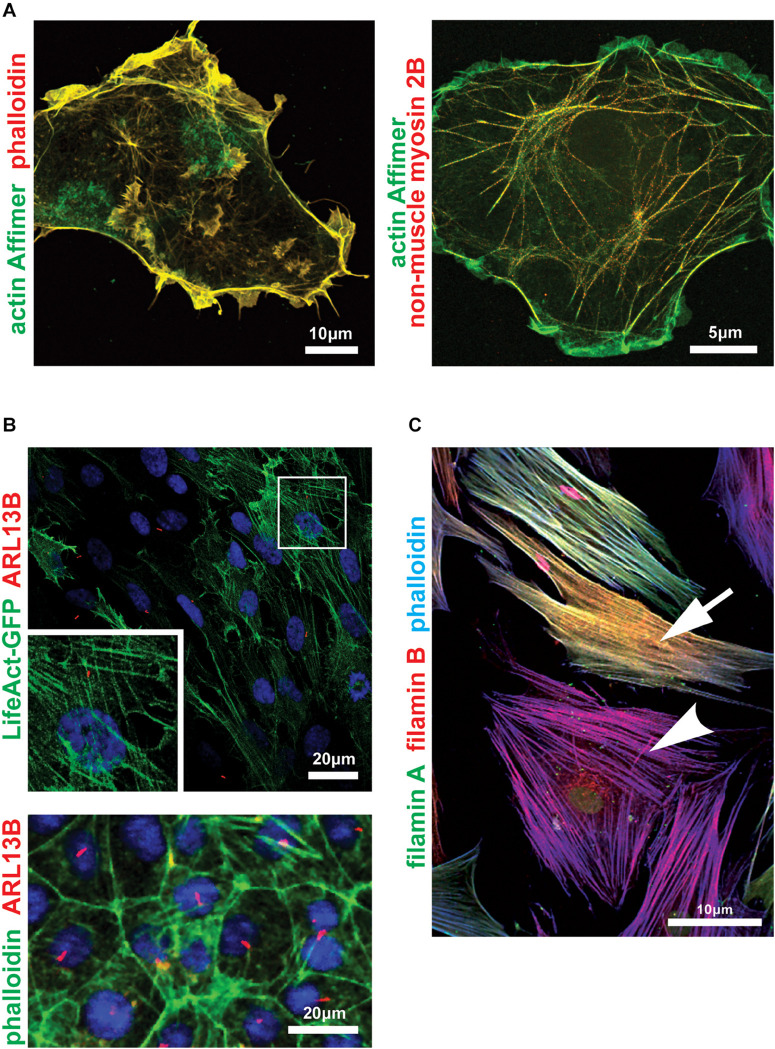
Imaging the actin cytoskeleton and actin-binding proteins within cells. **(A)** Zeiss Airyscan microscopy images of COS-7 cells probed with an actin-binding artificial non-antibody binding protein (“Affimer”) biotinylated on the C-terminal cysteine and visualized with fluorescent streptavidin (green). Cells were co-stained with fluorescent phalloidin (left panel; red) or antibody against non-muscle myosin 2B/MYH10 (right panel; red). Scale bars 10 or 5 μm, as indicated. **(B)** Confocal microscopy (upper panel) and high content imaging (lower panel) of ciliated serum-starved mouse inner medullary collecting duct (mIMCD3) cells expressing LifeAct-GFP (upper panel; green) or probed with AlexaFluor 488-phalloidin conjugate (lower panel; green). Primary cilia are marked by ARL13B (red). Frame indicates magnified inset showing detail of actin stress fibers in the upper panel. Scale bars = 20 μm. **(C)** Confocal microscopy image of human female dermal fibroblasts heterozygous for the *FLNA* frameshift mutation c.1587delG, p.(K529Nfs*40) (Adams et al., 2012) probed with antibodies for the actin-binding proteins filamin A (green) and filamin B (red), and counterstained with phalloidin-AlexaFluor633 conjugate (blue). The *FLNA* gene is carried on the X chromosome and random X-inactivation in different cells results in either haploinsufficiency (yellow-colored cell; indicated by arrow) or complete loss of filamin A protein (magenta-colored cell; arrowhead). Scale bar = 10 μm.

Phalloidin is a toxin originally obtained from the death cap mushroom (Lynen and Wieland, 1938) and is mainly used to label F-actin but it also acts to stabilize filaments (Wieland, 1977). Its effectiveness as a toxin, namely its strong specific binding to F-actin (K_d_ 0.27 μM), is exactly what has made it a useful research tool. As previously mentioned, electron microscopy of actin initially focused on the use of myosin to “decorate” actin filaments to reveal their polarity (Begg et al., 1978). Antibodies to actin were created as early as 1979 (Lazarides and Weber, 1974). However, the strong evolutionary conservation of actin makes it a notoriously weak immunogen and so other detection methods were sought. Phalloidin binds F-actin with much greater affinity than G-actin making it ideal to visualize actin structures. However, binding also stabilizes F-actin up to seven actin subunits from the binding site (Visegrády et al., 2005), preventing depolymerization. Stable actin aggregates can form as a result of phalloidin treatment of live cells (Wehland et al., 1977), so the stain must be used at very low concentrations to prevent artifacts. It also preferentially stains the ends of actin structures and might not stain all F-actin structures *in vivo* (Visegrády et al., 2005). Those caveats, plus its slow dissociation rate from F-actin and the need to microinject it into live cells, mean that phalloidin is instead most often used to stain fixed preparations where it remains the gold-standard actin stain (Figures 2A–C). Despite this, use of phalloidin, either as a directly labeled stain or as an agent applied to stabilize actin filaments, allowed the first visualizations of individual actin filaments (Yanagida et al., 1984; Honda et al., 1986). Observation of individual actin filaments and their dynamics without the interference of phalloidin was only possible with the use of alternative imaging techniques such as total internal reflection fluorescence microscopy (Fujiwara et al., 2002).

Actin-green fluorescent protein (GFP) fusion proteins have been used as an alternative to phalloidin in early live-staining studies of the actin cytoskeleton. However, GFP-tagged actin monomers polymerize less efficiently than endogenous actin (Yamada et al., 2005) and have therefore been associated with altered actin structure and dynamics (Spracklen et al., 2014). Actin-binding domains fused to GFP have been used to stain live cells with more success. These have utilized actin binding domains obtained from moesin in *Drosophila melanogaster* (Edwards et al., 1997), utrophin in *Xenopus laevis* (Burkel et al., 2007) and Abp120 and LimE in *Dictylostelium discoideum* (Pang et al., 1998; Bretschneider et al., 2004). These detection reagents have large domains and require transfectable cells, but can also affect F-actin structures and cell mechanics. Most recently, the much smaller “LifeAct” actin-binding domain, derived from the initial 17 amino acids of the yeast actin binding protein 140 (Abp140) (Riedl et al., 2008), has also been used for the reported ubiquitous visualization of F-actin (Lemieux et al., 2014; Figure 2B). However, like other proteins that bind and detect actin, LifeAct has also been reported to alter actin filament arrangements and dynamics in a dose-dependent manner (Courtemanche et al., 2016; Flores et al., 2019) and strong germline *in vivo* expression in fruit flies has been shown to cause sterility due to cytoskeletal defects (Spracklen et al., 2014). F-tractin is another small actin binding protein fragment, derived from rat inositol triphosphate 3-kinase (Johnson and Schell, 2009), but is less studied than LifeAct and also perturbs the actin cytoskeleton in *Xenopus* (Belin et al., 2014).

For all of these detection moieties, the fluorescent fusion protein and even the linker sequence used can alter the subset of F-actin labeled by the marker (Lemieux et al., 2014). More recently, chemically synthesized silicon-rhodamine (SiR) based probes such as SiR-actin have been reported (Lukinavičius et al., 2014). SiR probes exist in a zwitterion state, of which the fluorescent “on” state is promoted by interaction with polar protein surfaces whereas the non-fluorescent “off” state is promoted by aggregation or binding to hydrophobic surfaces (Lukinavičius et al., 2013). These probes are cell permeable and therefore do not require transfection or over-expression, but there are practical limitations because SiR only emits fluorescence in far-red and SiR-actin is derived from jasplakinolide so is likely to perturb actin polymerization and F-actin stabilization (Melak et al., 2017). Chromobodies have also been used to visualize actin dynamics in live cells (Rocchetti et al., 2014) and even whole organisms (Panza et al., 2015). Chromobodies are nanobodies, the antigen binding domain of single heavy-chain antibodies found in *Camelidae* (Hamers-Casterman et al., 1993), fused to fluorescent proteins such as GFP (Rothbauer et al., 2006). Chromobodies exhibit highly dynamic binding which allows the visualization of transient changes in actin dynamics without impairing actin movement or polymerization (Rocchetti et al., 2014). The most recent development in actin visualization are actin-binding artificial non-antibody binding proteins known as “Affimers” (Lopata et al., 2018; Figure 2A), although these have been shown to bind less well to dynamic actin structures. eGFP-Affimers may be more suitable for specialist applications, for example super-resolution microscopy or occasions when methanol fixation is required. Alternatively, they could be used to stain a specific subset of the cellular actin with further screening to identify more specific Affimers.

## Cilia and the Actin Cytoskeleton

The close associations between cilia and the actin cytoskeleton are clear from the outset of ciliogenesis, with the requirement of the actin cytoskeleton for centrosome/basal body migration and the anchoring of the cilia to the actin cytoskeleton by focal adhesion complexes (Antoniades et al., 2014). How these two mechanical constraints could modulate cilia length throughout ciliogenesis and disassembly (Mirvis et al., 2018) remains unclear. Since cilia are microtubule-based organelles, the traditional view has been that they do not contain actin. However, the curvature of the ciliary membrane clearly requires a cytoskeleton to maintain organization and an early report described indirect labeling of actin within the modified cilium of the photoreceptor using the myosin S1 sub-fragment (Chaitin and Burnside, 1989). More recently, two different forms of tagged actin were shown to localize to cilia in a ciliated cell-line, consistent with a role for F-actin in organizing the ciliary membrane into discrete sub-compartments known as nano-domains (Lee et al., 2018). Other reports have also highlighted the involvement of F-actin in both ciliary decapping (Phua et al., 2017) and ectosome excision (Nager et al., 2017) suggesting that actin might play a functional role within cilia, although it is notable that these studies did not identify possible actin-severing molecules that could reduce the length of actin filaments and mediate excision events. Direct detection of actin in cilia, at molecular resolution, has only recently been possible by using cryo-electron tomography (cryo-ET) preparations of primary cilia (Kiesel et al., 2020). This study identified bundles of actin close to the ciliary membrane and a structure resembling F-actin that intertwined with microtubules within the ciliary axoneme (Kiesel et al., 2020). These localizations were supported by immunofluorescence microscopy imaging with phalloidin and are consistent with a functional role of F-actin in maintaining the organization of the ciliary axoneme (Kiesel et al., 2020). Cryo-ET studies of the organization and molecular architecture of the primary cilium remain in their infancy, and there will undoubtedly be many further insights and surprises as cryo-ET workflows become integrated with correlated light microscopy studies of individual ciliary proteins.

F-actin has also been detected as a pool at the base of the cilium within the ciliary pocket (Molla-Herman et al., 2010). The ciliary pocket consists of a membrane invagination that is maintained by stable and dynamic actin filaments, in which the more dynamic actin is found at the distal end of the ciliary pocket in order to facilitate directional ciliary vesicle trafficking of ciliary cargo (Molla-Herman et al., 2010). This distal region undergoes clathrin-mediated endocytosis in response to phosphorylated dynein light chain Tctex-type 1 (DYNLT1) translocating to the transition zone, prior to S phase entry (Li et al., 2011; Saito et al., 2017). Phosphorylated DYNLT1 stimulates F-actin polymerization in the ciliary pocket by binding to actin and recruiting other regulators of actin polymerization (Saito et al., 2017), which is proposed to be one of the earliest steps in ciliary disassembly (Li et al., 2011).

## Actin and Regulation of Ciliogenesis Initiation and Cilia Length

Due to the high volume of traffic into and out of the cilium during normal cell homeostasis, ciliary maintenance and stability is essential for its function. The constant renewal of ciliary membrane proteins and transduction of signal requires functional IFT and delivery of cargo from the cytoplasm to the cilium. Currently, the mechanism for the delivery of ciliary membrane proteins is not fully understood. It is thought that ciliary cargo is arranged into ciliary-specific vesicles at the *trans* Golgi network. These marked vesicles are thought to be trafficked along the actin cytoskeleton (Kim et al., 2010, 2015; Cao et al., 2012) rather than microtubules and would therefore move by association with myosin motors (DePina and Langford, 1999).

The timing of actin dynamics and the exact role in ciliogenesis still requires further research, as it is poorly understood. Currently these dynamics are only modeled with global actin changes, which do not reflect the likely local and nuanced changes during normal ciliogenesis. In general, actin depolymerization, through either siRNA knock-down of actin regulators or pharmacological inhibition of actin polymerization with cytochalasin D, promotes ciliogenesis in all cell culture conditions (confluency and presence/absence of serum). Early studies have shown that pharmacological inhibition of actin polymerization promoted ciliogenesis and, furthermore, actin remodeling can transcriptionally control key negative regulators of ciliary disassembly Aurora A and Plk1 (Kim et al., 2015). This transcriptional control is mediated though YAP/TAZ-regulated Hippo signaling, which is activated by the actin remodeling (see below).

A large “druggable library” siRNA screen, that was published in 2010, sought to identify modulators of ciliogenesis and cilia length (Kim et al., 2010). The two main regulators that were further investigated were gelsolin (GSN), a positive regulator, and actin-related protein 3 (ACTR3), a negative regulator. Both are involved in the regulation of actin filament stabilization: GSN severs actin filaments and ACTR3 inhibits branching. Knock-downs of these genes, in parallel with cytochalasin D treatment suggested an important role of branched F-actin in modulating ciliogenesis, with dynamic or destabilized actin promoting ciliogenesis and increased cilia length. Cytochalasin D has also independently been shown to rescue ciliogenesis following siRNA knock-down of the important ciliary genes *CEP290*, *NPHP5*, and *IFT88* (Barbelanne et al., 2013). These studies suggested that destabilized F-actin increased vesicle trafficking, whereas stabilized F-actin would create a physical barrier to ciliary vesicle trafficking around the base of the cilium or centrosome during ciliogenesis (Kim et al., 2010).

A subsequent study identified LIMK2 and TESK1, two separate actin regulators exerting effects on cilia incidence (Kim et al., 2015). Both were linked to increased vesicle trafficking, which in-turn signaled changes in YAP/TAZ localization and Hippo signaling (Kim et al., 2015). However, both the screen and downstream work did not take into account the over-expression of Smo, a ciliary marker (Kim et al., 2010). When Smo is overexpressed it ectopically activates the Shh pathway, causing excessive GLI activation that is linked to cell cycle dysregulation (Valente et al., 2010; Kim et al., 2015), thus potentially confounding the assessment of the ciliary phenotypes in these studies. Nevertheless, these and previous studies indicate that actin cytoskeleton remodeling regulate both ciliogenesis initiation and cilia length.

To gain further mechanistic insight, further studies have assessed the possible role of non-muscle myosins in the remodeling of actin during ciliogenesis (Hong et al., 2015). A study by Rao et al. found that the myosin heavy chains Myh10 and Myh9 acted antagonistically to modulate ciliogenesis (Rao et al., 2014). Myh10-dependent actin dynamics were shown to regulate the correct localization of the pre-ciliary complex proteins Pcm1 and Cep290, thereby promoting the initiation of ciliogenesis. Interestingly, loss of cilia following Myh10 knock-down could be rescued by treatment with blebbistatin, an inhibitor with broad specificity for non-muscle myosin II that prevents acto-myosin contraction. These results suggest that ciliogenesis could be increased through the effects of acto-myosin contraction on actin cytoskeleton remodeling, leading to enhanced docking of the basal body at the apical cell surface during the early stages of ciliogenesis. This may be a separate, but not mutually exclusive, role to the proposed inhibitory effect of the actin cytoskeleton on ciliary vesicle transport. However, any role for ciliary vesicle transport would likely have a rapid and transient effect on the length of a pre-existing cilium, whereas acto-myosin contractility would affect basal body docking and overall cilia incidence over a longer time course of several hours. These differences in time-course for these potential mechanisms remain to be formally tested. Conversely, it is also unknown if actin remodeling can also promote cilia disassembly through facilitating transport away from the cilium.

## Actin at the Ciliary Tip

In comparison to proximal regions of the cilium, the ciliary tip has a different composition of proteins, including clathrins and actin, probably because it is a source of extracellular vesicles (ECV). The best investigated ECVs are from the retina outer segment, which allow recycling of opsins through endocytosis of ECVs by the retinal pigment epithelial cells (Young and Bok, 1969). In other cell types, ECVs are poorly characterized, but purified ECVs have been shown to contain ciliary proteins and transmembrane signaling molecules (Hogan et al., 2009; Nager et al., 2017). Therefore, ECVs may be used as a type of ciliary paracrine signaling between cells, or as a way for the cilium to rapidly regulate the levels of signaling proteins during signaling responses (Garcia et al., 2018). Recent research has revealed that disassembly of cilia occurs after an initial de-capping step generating ciliary ECVs. This de-capping is controlled by intraciliary F-actin and PI(4,5)P2, to bud off the tip of the cilium (Phua et al., 2017). Budding has been suggested to be a mechanism to quickly dispose of ciliary membrane proteins at the ciliary tip (Nager et al., 2017). This de-capping step then signals, through an unknown mechanism, to initiate full ciliary disassembly (Phua et al., 2017) through Aurora A activation and the direct phosphorylation of histone deacetylase 6 (HDAC6) (Pugacheva et al., 2007). This in turn deacetylates the ciliary axoneme, promoting ciliary disassembly. The full resorption of the remaining cilium is also poorly defined, and it is unknown if the cilium is resorbed from the base or is disassembled from the tip down. Further investigation of the roles of the kinesin-13 family proteins, kinesin family member 24 (Kif24) and kinesin family member 2A (Kif2a), is required to elucidate this process. In particular, Kif2a localizes to the subdistal appendages of the mother centriole, which are unlikely to play a direct role in depolymerization of axonemal microtubules (Miyamoto et al., 2015).

## Actin and Ciliary Stability

In addition to negative modulation of ciliogenesis and cilia length, actin has also been implicated in maintaining ciliary stability. Human and mouse mutant models of KDM3A, a multifunctional protein shown to have roles as a transcription factor for free actin, have reduced cellular actin levels and an associated increase in cilia (Yeyati et al., 2017). As there are decreased levels of actin around the base of the cilium in *Kdm3a* mutants, it was proposed that the loss of this physical gate would allow for an increase in IFT at the cilium, disrupting the balance of transport proteins (Yeyati et al., 2017). This dysregulation is further compounded by actin instability causing an increase in cilia length, further disrupting the balance and regulation of IFT (Yeyati et al., 2017).

## Role of Actin in Motile Cilia and Multiciliated Cells

Although there is compelling evidence for a functional role for actin destabilization in promoting ciliogenesis of primary cilia, in multiciliated cells there is support for the opposite effect (Pan et al., 2007). Multiciliated cells require basal bodies to both dock with the cell membrane and to establish polarity so that the cilia formed may beat in synchrony to establish fluid flow (Boisvieux-Ulrich et al., 1985; Mitchell et al., 2007). Ciliogenesis initiates with the formation of centrioles, which in multiciliated cells form *de novo* in an acentriolar fashion, i.e., they bud from an electron dense structure termed the deuterostome instead of duplicating from a mother centriole (Sorokin, 1968) and are trafficked to the apical cell surface to form basal bodies. This trafficking relies on association of the nascent centrioles with vesicles and the formation of an apical actin network (Boisvieux-Ulrich et al., 1990). The apical actin network both expands the membrane surface to allow for the docking of so many basal bodies, but also spaces them out across the surface of the cell membrane (Werner et al., 2011; Sedzinski et al., 2016). F-actin stabilization and an enriched actin web support the docking of basal bodies in mouse tracheal epithelial cells, thereby promoting ciliogenesis. This was induced by Forkhead box protein J1 (Foxj1), which promoted ras homolog family member A (RhoA) activity during ciliogenesis (Pan et al., 2007), a process that may be downstream of initial dynamic actin remodeling that allows centriole migration. WDR5 has been shown to be important in both establishing and maintaining the apical F-actin network in multi-ciliated cells and for the docking and anchoring of basal bodies at the apical cell membrane (Kulkarni et al., 2018). RhoA, Fmn1 (Sedzinski et al., 2017), phosphate loop ATPase Nubp1 (Ioannou et al., 2013), ezrin (Huang et al., 2003), Fak (Antoniades et al., 2014), FoxJ1 (Huang et al., 2003; Stubbs et al., 2008) and planar cell polarity proteins [including Disheveled (Park et al., 2008), Inturned and Fuzzy (Park et al., 2006)] have all been shown to be important in either establishing the apical actin network or for the trafficking of basal bodies to the apical cell membrane.

## Actin Binding Proteins, Ciliogenesis and Cilia Maintenance

Following on from the seminal large “druggable library” siRNA screen by Kim et al., which identified a number of actin binding proteins as modulators of ciliogenesis and cilia length (Kim et al., 2010), more recent studies have identified additional actin binding proteins that have novel and unexpected roles in ciliogenesis and ciliary maintenance. Here we discuss several actin binding proteins that have been studied in the most detail.

### Filamin A

Filamin A (FLNA, previously named FLN1 and ABP-280) is one of three proteins in the filamin family (Figure 2C). Filamins anchor the actin cytoskeleton to the cell membrane by crosslinking actin into networks in the cortical cytoplasm (Nakamura et al., 2011). They can also bundle actin into stress fibers (Hirata et al., 2007). FLNA is a ubiquitously expressed, 280 kDa phosphoprotein dimer that consists of three main domains, an N-terminal actin binding domain that itself contains two pairs of calponin homology (CH) domains, a rod domain, consisting of immunoglobulin-like repeats interrupted by two hinge regions and a C-terminal binding domain important for both self-dimerization and binding to other membrane receptors (Wade et al., 2020). Mutations in *FLNA* cause a variety of X-linked disorders (Wade et al., 2020). These include multiple malformation syndromes, congenital short bowel disorder, terminal osseous dysplasia, cardiac valvular dysplasia and periventricular heterotopia (Fox et al., 1998; Wade et al., 2020). Periventricular heterotopia (PH, OMIM #300049) describes the persistence of nodules of neurons lining the ventricular surface of the brain following their failure to migrate to the cerebral cortex (Fox et al., 1998). It results from hypomorphic or loss-of-function variants that cluster in the actin-binding domain, particularly within the CH domains (Parrini et al., 2006).

FLNA was first linked to centrosomal trafficking and initiation of ciliogenesis through the identification of a patient with both MKS and PH (Adams et al., 2012), who was found to have a mutation in *TMEM67* (c.2754_2756delCTT, p.I918_F919del), deleting two residues in the C-terminal cytoplasmic region shown to directly interact with FLNA. FLNA localizes at the apical cell surface in polarized cells, but also at the basolateral membrane (Adams et al., 2012), which is also consistent with the cellular localization of TMEM67 (also known as meckelin) at both the basal body/transition zone and in non-ciliary vesicles (Dawe et al., 2007). *In vitro* and *in vivo* studies showed that reduced or absent FLNA caused defects in basal body positioning and ciliogenesis, and aberrant remodeling of the actin cytoskeleton. Investigations using fibroblasts from a female with a heterozygous pathogenic frameshift in *FLNA* showed than the basal body positioning was maintained in the mid portion of the cell rather than at an apical position (Adams et al., 2012). Similar effects were shown in a null mouse model and for centrosomal positioning when using siRNA knockdown on mIMCD3 cells (Adams et al., 2012). Ciliogenesis was also impaired, but not abolished, in all models. Interestingly, abrogation of the interaction between TMEM67 and FLNA resulted in the disruption of RhoA activation and signaling, with probable downstream effects on non-canonical Wnt signaling and ciliary function (Adams et al., 2012).

More recently, it has been suggested that transforming acidic coiled-coil protein 3 (TACC3) may compete with TMEM67 (meckelin) for FLNA binding (Qie et al., 2020). TACC3 is up-regulated in prostate cancer (Qie et al., 2020) and it promotes cell growth and differentiation in a number of other cancers (Lauffart et al., 2005; He et al., 2016; Jiang et al., 2016). Primary cilia are often reduced or entirely lost in cancerous cells (Schraml et al., 2009; Gradilone et al., 2013; Menzl et al., 2014), but knockdown of TACC3 resulted restoration of cilia in a human prostate cancer cell-line (Qie et al., 2020). It is therefore possible that over-expression of TACC3 competes with the TMEM67-FLNA interaction, thereby restricting centrosome migration to the apical cell surface (Qie et al., 2020).

### RhoA

RhoA is the activator of ROCK, a key actin remodeling regulator that is responsible for the formation of stress fibers (Ridley and Hall, 1992). RhoA has been shown to contribute to the molecular pathology of ciliopathies: increased RhoA levels were observed in dermal fibroblasts from ciliopathy patients with *TMEM216* mutations (Valente et al., 2010). Independently, RhoA has also been shown to mislocalize in patients with JBTS syndrome (specifically caused by *TMEM237* mutations), and patient fibroblasts had increased actin stress fibers (Huang et al., 2011).

The cellular phenotypes observed in many ciliopathies are suggested to be due to defects of the actin cytoskeleton (Valente et al., 2010; Adams et al., 2012; Hernandez-Hernandez et al., 2013). A study into disease mechanisms of Bardet-Biedl syndrome observed that *Bbs4*- and *Bbs6*-deficient renal epithelial cells derived from mutant mice had decreased cilia incidence, associated with increased focal adhesions and abnormal actin stress fibers. These aberrant changes in the actin cytoskeleton were ascribed to highly up-regulated RhoA expression (Hernandez-Hernandez et al., 2013). Since RhoA-GTP is a direct activator of ROCK, increased ROCK activity would lead to increased stress fiber formation and F-actin stabilization. As a consequence, when *Bbs4*- and *Bbs6*-deficient cells were treated with Y27632, a non-specific inhibitor of the ROCK and ribosomal S6 kinase (RSK) families, cilia incidence was rescued. This observation supports the hypothesis that the actin cellular phenotype is a significant cause of the loss of cilia in the *Bbs4* and *Bbs6* mouse mutants, although it does not exclude the effect of other signaling pathways.

### Synaptic Nuclear Envelope Protein 2

Synaptic nuclear envelope protein 2 (SYNE2) is a member of the nuclear envelope spectrin repeat (nesprin) family of proteins that comprises four members (Rajgor and Shanahan, 2013). Previously named nesprin-2, SYNE2 is a modular protein with N-terminal paired calponin homology (CH) domains, a C-terminal Klarsicht/ANC-1/Syne homology (KASH) transmembrane domain that acts as a nuclear envelope targeting motif (Zhen et al., 2002), and a central extended spectrin repeat rod domain thereby tethering the nucleus to the cytoskeleton. SYNE2 binds to cytoplasmic F-actin directly through its actin-binding CH domains, but also itself binds FH1/FH2 domain-containing protein 1 (FHOD1) and fascins (FSCN1, FSCN2, FSCN3) which also bind actin (Chang et al., 2015). Collectively, these contribute to the linker of nucleoskeleton and cytoskeleton (LINC) complex, which associates the nuclear membranes with the cytoskeleton, including actin, microtubule filaments, intermediate filaments, centrosomes and cytoplasmic organelles.

SYNE2 was first linked to centrosome trafficking and early ciliogenesis, when it was shown that a splice variant lacking the KASH domain interacts with two MKS proteins (MKS1 and TMEM67) that are required for centrosome migration and ciliogenesis (Dawe et al., 2009). Depletion of either SYNE1 or SYNE2 caused defective centrosome migration during early cell polarization, leading to defects in early ciliogenesis (Dawe et al., 2009). Following depletion or mutation of TMEM67, SYNE2 localized at actin stress fibers and RhoA signaling aberrantly increased, implicating SYNE2 in the control of the actin cytoskeleton during cell polarization and early ciliogenesis (Dawe et al., 2009). Subsequent work in a ciliated cell-line with a gene-edited *SYNE2* knockout has supported a functional role for SYNE2 during ciliogenesis (Falk et al., 2018).

SYNE2 has also been shown to interact with pericentrin (PCNT) (Falk et al., 2018). PCNT is a component of pericentriolar material (present at the base of cilia) and is a large coiled coil protein which localizes to the centrosome via a PACT motif (Miyoshi et al., 2006). It also serves as a protein scaffold and contributes to mitotic spindle organization and ciliogenesis (Jurczyk et al., 2004; Zimmerman et al., 2004). SYNE2 and PCNT have been shown to co-localize at the connecting cilium of the photoreceptor cells of the retina (Mühlhans et al., 2011; Falk et al., 2018). Gene-edited SYNE2 cells, lacking the PCNT binding domain, also had shorter cilia compared to other mutant SYNE2 cells with the domain, suggesting that the interaction may be required for delivery of ciliary proteins to the ciliary base (Falk et al., 2018).

### PCARE and Retinal Outer Segments

An example of the importance of the actin cytoskeleton in the role of cilia within specialized cell types has been highlighted in a recent paper on photoreceptors (Corral-Serrano et al., 2020). The connecting cilium between the inner and outer segments of photoreceptors is analogous to a typical ciliary transition zone, with the outer segment effectively acting as a modified primary cilium. Within every photoreceptor outer segment, around 10% of the light-sensing, opsin-containing discs are shed daily at the apical end (Young, 1967). New discs form at the base and move through the length of the outer segment until their shedding and destruction. The mechanism by which new discs are formed has been shown to involve expansion of the ciliary plasma membrane at the point at which the connecting cilium meets the base of the outer segment, but the proteins involved in driving the expansions have only recently been identified.

**FIGURE 3:**
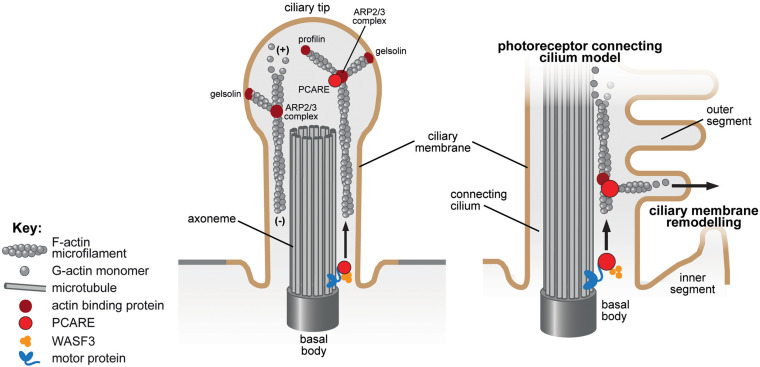
Ciliary membrane remodeling of the actin-binding protein PCARE in the primary cilium and photoreceptor connecting cilium. The proposed role of PCARE in the cilium **(left panel)** and in the specialized cilium of the photoreceptor outer segment and connecting cilium **(right panel)** are indicated. PCARE (red) has been shown to interact with actin-binding proteins (dark red symbols) associated with *de novo* F-actin network assembly including the ARP2/3 complex, gelsolin, profilin and WASF3 (orange). PCARE in mice localizes to the basal body, the microtubules of the connecting cilium and extends into the newer outer segment discs (Corral-Serrano et al., 2020). Overexpression of PCARE in hTERT RPE-1 cells resulted in WASF-3 translocating from F-actin to the cilium and induced expansion of the ciliary membrane to form a bulbous tip **(left panel)**. PCARE is a retinal specialist protein that facilitates actin remodeling via recruitment of WASF3 to induce membrane remodeling and expansion that produces new outer segment discs for photo-sensation in the photoreceptor cell (Corral-Serrano et al., 2020).

Photoreceptor cilium actin regulator (PCARE, previously named C2orf71; Figure 3) was first identified when it was shown to be mutated in a subset of retinitis pigmentosa patients (RP54) (Collin et al., 2010; Nishimura et al., 2010). Its expression is predominantly retinal and it contains a W2 domain thought to bind to actin filaments (Corral-Serrano et al., 2020). Through interaction studies, PCARE has been shown to interact with retinal disease proteins associated with the centrioles of the centrosome and basal body as well as actin proteins associated with *de novo* F-actin network assembly including ARP2/3, ENAH, gelsolin, profilin 1, profilin 2, MYL12A, MYL12B, LIMA1, filamin A and WASF3 (Corral-Serrano et al., 2020). PCARE in mice localizes to the basal body, the microtubules of the connecting cilium and extends into the newer outer segment discs (Figure 3) and *Pcare*^–/–^ mice have disrupted OS disc stacking (Corral-Serrano et al., 2020). Overexpression of PCARE in hTERT RPE-1 cells resulted in WASF-3 translocating from F-actin to the cilium and induced expansion of the ciliary membrane to form a bulbous tip (Corral-Serrano et al., 2020). Culture of retinal organoids showed that such expansions formed the new outer segment discs. Inhibition of actin polymerization by cytochalasin D or lantrunculin B treatment also reduced the formation of the ciliary tip expansions, as did siRNA knockdown of ARP2 in mIMCD3 cells (Corral-Serrano et al., 2020). Similarly, RP-causing mutant PCARE had the same localization as wild-type protein but failed to cause the same expansion of the ciliary tip, interpreted as a failure to induce actin remodeling (Corral-Serrano et al., 2020). It therefore appears that PCARE is a retinal specialist protein that facilitates actin remodeling via recruitment of WASF3 to induce membrane expansion that produces new outer segment discs for photo-sensing.

### Leucine Zipper Protein 1

Another important actin binding protein implicated in ciliogenesis is leucine zipper protein 1 (LUZP1) (Wang and Nakamura, 2019). In cycling cells, LUZP1 localizes to the pericentriolar matrix at the proximal end of each centriole, the actin cytoskeleton and the midbody (Bozal-Basterra et al., 2020). In cell cycle-arrested cells, LUZP1 localizes to the basal body and to the ciliary axoneme. LUZP1 has been shown to bind directly with actin but also through interactions with filamin A (Hein et al., 2015; Wang and Nakamura, 2019) and ARP2 (Hein et al., 2015). A functional role for LUZP1 in ciliogenesis has been suggested through studies of the molecular etiology of Townes-Brocks syndrome (TBS1 MIM #107480). In TBS1 cells, cilia are longer and occur at a higher incidence than in wildtype cells. TBS1 is caused by mutations in the spalt-like transcription factor 1 gene (*SALL1*) (Kohlhase et al., 1998). The encoded protein, SALL1, normally interacts with and removes negative regulators of ciliogenesis (CCP110 and CEP97) from the mother centriole, thereby promoting ciliogenesis. However, truncated mutant forms of SALL1 that cause TBS1 appear to interact with LUZP1 (Bozal-Basterra et al., 2018). This interaction causes LUZP1 degradation through the ubiquitin proteasome system, since inhibition of the proteasome in TBS1 cells lead to accumulation of LUZP1 (Bozal-Basterra et al., 2020). Loss of LUZP1 results in reduced actin polymerization, increased cilia incidence and length, and increased Shh signaling (Bozal-Basterra et al., 2020; Gonçalves et al., 2020). In contrast, over-expression of LUZP1 in TBS1 cells resulted in increased levels of F-actin and reduced ciliogenesis to normal levels, suggesting that LUZP1 is a potential therapeutic target for the treatment of TBS1 (Bozal-Basterra et al., 2020).

## Proximity Labeling Studies

Studies utilizing proximity labeling to identify ciliary proteins have reported varied results for detection of actin binding proteins (Mick et al., 2015; Kohli et al., 2017). One study revealed a surprising number of actin binding proteins within cilia (Kohli et al., 2017). Kohli et al. used enzyme-catalyzed proximity labeling with an engineered ascorbate peroxidase (APEX2) that was specifically targeted to the ciliary membrane (Kohli et al., 2017). Actin-binding proteins that were identified in close proximity to the ciliary membrane included activins, tropomyosins, coronin, ezrin, gelsolin, cortactin, utrophin, drebrin-like protein, and, most abundantly, alpha-activin and filamin A (Kohli et al., 2017). However, it was unclear if proximity labeling was also tagging actin-binding proteins during trafficking of the construct at the ciliary pocket which would argue against a specific role for these proteins within cilia. Paradoxically, levels of these actin binding proteins increased in the ciliary compartment after cells were treated with the actin-depolymerizing agent cytochalasin D (see above). An earlier study, utilizing APEX that was specifically targeted to the ciliary lumen using nephrocystin 3, failed to detect actin binding proteins within the cilium (Mick et al., 2015).

Actin depolymerization may therefore trigger the recruitment of actin-binding proteins to the cilium (Kohli et al., 2017), but it remains unclear how this leads to axonemal extension and cilium elongation, and whether actin-binding proteins have a direct role in this process. It is possible that actin de-polymerization causes the non-specific release of the pool of proteins normally bound to F-actin at the ciliary pocket and subsequent ectopic release into the ciliary compartment.

## Pharmacological Inhibition of Actin Remodeling

Interest in modification of the actin cytoskeleton as a possible treatment for ciliopathies grew from the finding that positive regulators of actin polymerization exerted negative effects on ciliogenesis and vice versa (Kim et al., 2010). Kim et al. (2010) also found that cytochalasin D treatment, which prevents actin polymerization, significantly rescued the ciliogenesis defect in hypomorphic IFT88 mutant cells (*Ift88*^orpk/orpk^). Cytochalasin D is unsuitable for treatment of ciliopathies in whole organisms because of the effects of global inhibition of actin polymerization, that include failure of cytokinesis, aneuploidy, cell cycle arrest and apoptosis, as well as off-target effects of the inhibitor. However, other inhibitors that target actin modulators implicated in ciliogenesis have been suggested to be more suitable for pharmacological intervention and treatment (Kim et al., 2010).

Drugs that act on actin binding proteins and increase cilia incidence could also be valuable for diseases of spontaneous epithelial-mesenchymal transition and uncontrolled cell division such as cancer (Izdebska et al., 2018). Specific molecules targeting some actin binding proteins have been designed, specifically against ARP2/3 (Nolen et al., 2009), non-muscle myosin II (MYH9/10) (Saitoh et al., 1987; Straight et al., 2003) and neuronal WASP (Peterson et al., 2004). However, to date, undesirable effects of such general inhibition have precluded these compounds from clinical practice (Chánez-Paredes et al., 2019). Tissue-specific inhibition through the specific targeting of an actin binding protein such as a PCARE or a SYNE2 isoform expressed only in brain and kidney could be a more specific therapeutic strategy to modulate actin conditions in tissues that express ciliopathy phenotypes.

## Identification of Therapeutics for Cystic Kidney Disease

Several drugs screens have been completed to identify compounds that could be used to modulate the actin cytoskeleton and increase ciliogenesis (Table 1). These screens have focused on the kidney since polycystic kidney disease is relatively common [4 in 10,000 in Europe (Willey et al., 2017)] and cystic kidney disease is a common feature of many ciliopathies (Habbig and Liebau, 2015). Practically, kidney disease presents later than many other symptoms of ciliopathies, such as retinitis pigmentosa or developmental brain anomalies and therefore there is a therapeutic window *ex utero* where a drug could be given. In addition, cell lines of kidney origin (e.g., mIMCD3) are used as a standard model in cilia research and can be easily assayed using high-throughput microscopy (Wheway et al., 2015). Patient cells can also be isolated through collection and culture of urinary renal epithelial cells (URECs) from urine or through collection of kidney cysts for more focused screening of hit compounds (Loghman-Adham et al., 2003; Ajzeberg et al., 2015). Future screens are likely to test compounds on kidney organoids due to advances in gene editing and directed differentiation of induced pluripotent stem cells (iPSCs). IPSCs can be engineered to carry specific mutations of interest using CRISPR-Cas9 editing and kidney organoids can be produced from iPSCs in less than a month of differentiation (Takasato and Little, 2017).

**TABLE 1:** Drug screens to identify compounds that modulate the actin cytoskeleton and increase ciliogenesis.

Cell line/origin	Mutational model	Cell culture	Stimulation	Drug library	Methodology	Output (assay markers)	Validation	Hit categories	Specific Hits	References
CFPAC-1	wild-type	2D	none	Pharmakon 1600	10 μM with 2 × 4 day exposure	% ciliated cells (acetylated alpha-tubulin)	assays of hit compounds in four other cancer cell-lines	118 hits: 49 glucocorticoids, fibrates or other nuclear receptor modulator; 14 neurotransmitter modulators; other include ion channel modulators, tyrosine kinase inhibitors	cilia incidence: clofibrate, gefitinib, sirolimus; cilia length: imexon, clofibrate, xylazine	PMID:26862738
mIMCD3	*Pkd1*^–/–a^	3D	forskolin treatment	L1200 SelleckChem (273 kinase inhibitors)	0.1 and 1 μM forskolin for 72 h	cyst size (F-actin and nuclei)	dose response assays of hit compounds	inhibitors targeting mTOR, Aurora A kinase, CDK, IGF-1R, and dual mTOR/PI3K inhibitors	torin 1 and torin 2	PMID:28644734
MEK	*Pkd1*^–/–^, *Pkd1*^+/–^ and wild-type	2D screen, 3D validation	none	NIH Pharmaceutical Collection (NPC) and other collections (8814 compounds)	various concentrations over 48 h, followed by comparison between different genotypes	cell proliferation (fluorescence assay), ATP levels (luminescence assay)	re-testing fresh hit compounds, dose response and 3D cyst assays in murine cell; assays in human ADPKD cells and normal kidney cells	155 hits from 2D screen: HMG-CoA reductase, HSP90, tubulin depolymerization and other inhibitors; 109 hits validated in 3D models; 21 hits validated in human ADPKD cells	3D models: epothilone A, GSK-269962A, 5-azacytidine, tosedostat; human ADPKD cells: gemcitabine, niclosamide, cerivastatin	PMID:32144367
mIMCD3	*Pkd1*^–/–b^	3D	none	Cayman Chemicals #10505 (155 kinase inhibitors)	6 day treatment, various concentrations 1-33 μM	% cord/tubule vs. cysts (F-actin)	treatment of conditional AhCre; *Pkd1*^flox/flox^ mouse model with Y-27632^c^	5 hits: ROCK inhibitors	Y-27632, HA-1077, H-89, (S)-H-1152, (S)-Glycyl-H-1152	PMID:29891559

*CFPAC-1, ductal pancreatic adenocarcinoma cell-line; mIMCD3, murine inner medullary collecting duct kidney epithelial cell-line; MEK, murine embryonic kidney cells derived from collecting duct and post-natal proximal tubule cells. ^a^Engineered using lentiviral transfection. ^b^Engineered using CRISPR-Cas9 gene editing. ^c^No other specific validation of hit compounds; further experiments focused on the RhoA-YAP-cMyc signaling pathway.*

Of the tens of drugs screens carried out, one screen of 1600 compounds identified 118 hits that restored cilia expression in the CFPAC-1 pancreatic cancer cell line (Khan et al., 2016). The authors stated that many of these compounds affected levels of cAMP, calcium or other ions and highlighted glucocorticoids as the main drug class identified. Notably, glucocorticoids can both stimulate and inhibit Smo accumulation in cilia during Sonic Hedgehog signaling (Wang et al., 2012). Other hits identified included neurotransmitter regulators and ion channel modulators, as well as compounds that were previously identified to increase cilia incidence, cilium length or ciliary beat frequency in multi-ciliated cells.

Several other small molecule screens have highlighted inhibition of ROCK, amongst many other pathways, as a potential therapeutic for cystic kidney disease. One study screened 273 kinase inhibitors in a forskolin-stimulated 3D cyst assay using an IMCD3 cell line expressing a short hairpin RNA targeting polycystin 1 transcripts and identified thiazovivin (a non-selective ROCK inhibitor) as a significant hit (Booij et al., 2017). Hits also included inhibitors of IGF-1R, HER2, CHK1/2, PLK1, CDKs and AURKA but the largest category was for inhibitors of mTOR (Booij et al., 2017). The identification of inhibitor hits for so many different pathways may reflect the importance of proliferation in cyst growth and highlights that the use of these inhibitors is unlikely to be a useful treatment, due to off-target effects with other kinases and the effects of global inhibition of these pathways on the organism as a whole.

One recent screen assessed the effects of 8,814 compounds, mostly with proven anti-neoplastic effects, on autosomal dominant polycystic kidney disease type 1 (ADPKD1) adult and fetal cell models (Asawa et al., 2020). This screen looked for compounds that reduced cell viability of polycystin 1 (*PKD1*) null lines but not of wildtype cells. The authors then rescreened a selection of compounds for their effects on a 3D cyst culture (unstimulated by cAMP). Although some compounds had no effect on cell viability in 2D, they had significant effects on cyst growth in the 3D model (Asawa et al., 2020). Compounds that affect cell viability or proliferation are unlikely to be effective treatments for all stages of renal cystic disease. However, a number of compounds had no effect on cell viability but nevertheless still reduced cyst size. These included several compounds known to affect microtubule dynamics, including a DNMT1 inhibitor, an M1 aminopeptidase inhibitor and a ROCK inhibitor (GSK 269962). These are promising compounds for future studies of clinical efficacy and potential repurposing.

In support of the potential repurposing of known ROCK inhibitors, one study demonstrated rescue of cord and tubule formation whilst reducing cyst formation in the cystogenic *Pkd1* knock-out 3D kidney cell-line model (Cai et al., 2018). This study identified that *Pkd1* knock-out caused an increase in RhoA-ROCK-MLC signaling, leading to downstream activation of the Hippo signaling pathway. Screening of a panel of 155 kinase inhibitors identified five hit compounds, of which all were ROCK inhibitors, including a selective ROCK2 inhibitor, which suggests that pharmacological targeting of ROCK2 rather than ROCK1 may have therapeutic benefit. Further investigation of the efficacy of these drugs using more physiologically relevant, slower developing models of cystic kidney disease, is warranted. It is also important to test the drugs using models that had already developed cysts rather than treating models prophylactically, before cysts had appeared, to see whether ROCK inhibitors can ameliorate existing disease.

Aside from screens, ROCK inhibitors have also been shown to restore cilia in a Rho GTPase activating protein (GAP) mutant mouse model (*Arhgap35*^D34/D34^) that had reduced cilia incidence and length (Stewart et al., 2016). This Rho-GAP mutant had increased RhoA activation as a result of reduced intrinsic hydrolytic activity due to inactivation of ARHGAP35. The mutant mouse model also displayed a glomerulocystic kidney phenotype, thus linking RhoA hyperactivity and failures in ciliogenesis to cystogenesis (Stewart et al., 2016). Further investigation identified a reduction in cilia within the proximal tubule during development, resulting from a failure in axoneme elongation rather than basal body migration or docking (Stewart et al., 2016). The authors concluded that local negative regulation of Rho-GAPs is required at the ciliary base to enable axoneme extension (Stewart et al., 2016). A more recent study reported that dysregulation of RhoA signaling at the centrosome, mediated by ARHGAP35, led to increased activation of ROCKs in mutant or null *PKD1* cells (Streets et al., 2020). The authors found that treatment of *PKD1* patient cells and an inducible, kidney specific *Pkd*^–/–^ mouse model with the ROCK inhibitor hydroxyfasudil reduced cyst size over 7 days of treatment (Streets et al., 2020). Collectively, these findings call for the assessment of the effects of different ROCK inhibitors on cyst growth and development in human cell and animal models. Assessment of the relative contributions of F-actin stabilization and acto-myosin contraction to ciliogenesis and cilia maintenance over different time periods will be key to understanding how ROCK inhibitors modulate cilia. Parallel investigations should also assess the effect on ROCK inhibition on ciliary-mediated signaling functions.

## Conclusion and Future Directions

It is now recognized that the centrosome acts as both a microtubule and actin organizing center, and therefore that the actin cytoskeleton and ciliogenesis are intrinsically linked. It is also clear that the actin cytoskeleton exerts multiple effects on ciliogenesis, cilia maintenance, signaling and function, making it an important potential drug target. The large number of actin-binding proteins that are involved in actin dynamics, often in cell-specific processes, means that targeting can be potentially fine-tuned depending upon the cell type. Greater understanding of actin remodeling will come from improved actin detection and visualization of highly dynamic remodeling events, including the use of super-resolution microscopy and the use of proximity labeling studies to identify novel actin binding proteins.

In the next stages of this research, additional screens of candidate drugs or small molecules should be completed, using a number of different models of cystic kidney disease, with different genetic aberrations and backgrounds and with treatment at different disease stages. These studies will be required to discern whether inhibitors of ROCK or other targets that modulate the actin cytoskeleton may have potential clinical efficacy in the treatment of cystic kidney diseases. These screens will also help to determine the points at which disease can be ameliorated and which disease stage would be most amenable to the effects of each drug. RNA expression profiling of ADPKD and *Pkd1*^–/–^ kidney cysts compared with healthy kidney tissue supports the targeting of the RhoA-YAP-cMyc axis (Cai et al., 2018), but wider profiling could be useful to identify other pathways to target at specific timepoints in cystogenesis or for particular genotypes. Additional profiling could also be useful to narrow the pathways already identified, which include regulation of calcium ions, cAMP, MAPK, JAK, SRC, STAT, Wnt, and mTOR (Lemos and Ehrlich, 2018). It will be interesting to determine if activation of the RhoA-YAP-cMyc signaling axis is also observed in cysts from other disease models with mutations in other genes. However, the renal sub-location where cysts develop is partially dependent on genotype (Gascue et al., 2011), and it is therefore likely that pathomechanisms will depend on the genetic etiology of the cystic kidney disease. Different disease types may therefore require different drug therapies, although the therapeutic targeting of a single actin modulator such as ROCK may provide a common strategy that is suitable for several disease classes. It is notable that several newly developed ROCK inhibitors are experimental drugs currently (October 2020) in phase II clinical trials for treatment of chronic graft-versus-host disease, idiopathic pulmonary fibrosis and psoriasis. It will be interesting to determine if these drugs could be re-purposing for treatment of cystic kidney disease.

## Author Contributions

CS, AL, and CJ drafted the manuscript and figures. All authors gave final approval and agreed to be accountable for all aspects of the work.

## Conflict of Interest

The authors declare that the research was conducted in the absence of any commercial or financial relationships that could be construed as a potential conflict of interest.
