# Supplementary material for: Primary Cilia, Ciliogenesis and the Actin Cytoskeleton: A Little Less Resorption, A Little More Actin Please
Source: Front Cell Dev Biol. 2020 Dec 17;8:622822. doi: 10.3389/fcell.2020.622822 (PMC7773788; doi:10.3389/fcell.2020.622822)
Supplement: Supplementary file 1 [file Table_1.DOCX]

Supplementary Material

Primary cilia, ciliogenesis and the actin cytoskeleton: a little less resorption, a little more actin please.

Claire E.L. Smith, Alice V.R. Lake and Colin A. Johnson^*^

*** Correspondence:** Colin A. Johnson: c.johnson@leeds.ac.uk

| **Protein family** | **Actin binding / ciliary protein role** | **Exemplar protein(s)** | **Gene symbol** | **Other names & symbols** | **UniProt ID** | **Summary of function** | **Key publications (reviews)** |
| --- | --- | --- | --- | --- | --- | --- | --- |
| SPIRE | actin nucleation | SPIRE type actin nucleation factor 1 | *SPIRE1* | spire homolog 1 (Drosophila) | Q08AE8 | nucleation factor associated with (-) end of actin filaments; mediates intracellular transport along actin fibers | PMID: 11747823, PMID: 18042452 |
| formins | actin nucleation | formin 1 | *FMN1* | formin homology proteins | Q68DA7 | associated with (+) end of actin filaments; most are Rho-GTPase effector proteins mediating actin polymerization | PMID: 12775772, **29742020** (31862221, **31913455**) |
|  | actin nucleation, capping & cross-linking | formin homology 2 domain containing 1 | *FHOD1* | FHOS | Q9Y613 | required for the formation of F-actin structures such as stress fibers; activated by Rho signaling | PMID: 31657439 |
| actin related protein complex (ARP2/3) | actin nucleation, branching, depolymerization & severing | actin related protein 2 & 3 | *ACTR2, ACTR3* | ARP2 & ARP3 | P61160, P61158 | complex binds to existing filaments, initiates branching of new filaments; the ARP2/3 subunits resemble actin monomers that act as nucleation sites | PMID: 16990851, **11721045** |
| ADF / cofilin | actin depolymerization & severing | cofilin 1 | *CFL1* | non-muscle cofilin | P23528 | regulates assembly & disassembly of actin filaments; depolymerizes filaments at (-) end; severs filaments to create new (+) ends; phosphorylated & activated by LIMK in response to Rho signaling | PMID: 26704451, 23406901 |
|  |  | destrin | *DSTN* | actin depolymerizing factor (ADF) | P60981 | regulates assembly & disassembly of actin filaments; depolymerises filaments at (-) end; severs filaments & binds G-actin monomers | PMID: 30692249 |
| Wiskott-Aldrich syndrome proteins (WASP) | actin nucleation & branch formation | WASP actin nucleation promoting factor | *WAS* | Wiskott-Aldrich syndrome 1; thrombocytopenia 1 (X-linked) | P42768 | binds & activates ARP2/3 complex; required for efficient actin polymerization; effector protein for Rho-GTPases; WASP depletion promotes ciliogenesis | PMID: 29945904 |
|  |  | WASP family member 3 | *WASF3* | WAS protein family, member 3 (WAVE3), SCAR3 | Q9UPY6 | effector protein for Rho-GTPases during actin cytoskeleton remodeling; binds to retinal protein PCARE to induce ciliary membrane expansion | PMID: 32312818 |
| spectrin proteins | actin cross-linking & bundling | actinin alpha 1 | *ACTN1* | alpha actinin | P12814 | cross-linking protein that anchors F-actin to intracellular structures (e.g. stress fibers, filopodia) | PMID: (27698030) |
|  | actin cross-linking | spectrin repeat-containing nuclear envelope protein 2 | *SYNE2* | nuclear envelope spectrin repeat-2 (nesprin-2), nucleus and actin connecting element (NUANCE) | Q8WXH0 | component of the LINC complex; binds to cytoplasmic F-actin through FH1/FH2 domain-containing protein 1 (FHOD1) and fascins; required for nuclear & centrosome positioning; SYNE2 implicated in early ciliogenesis | PMID: 19596800, 30054381, 30141036 |
|  | actin binding | utropin | *UTRN* | DMDL, DRP, DRP1 | P46939 | may anchor the cytoskeleton to the plasma membrane | PMID: 15837517 |
| filamins | actin cross-linking | filamin A | *FLNA* | FLN1, actin binding protein 180 (abp180) | P21333 | anchor the actin cytoskeleton to the cell membrane by cross-linking actin into networks; bundle actin into stress fibers; FLNA mediates basal body positioning during ciliogenesis | PMID: 22121117 |
| fascins | actin cross-linking & bundling | fascin actin-bundling protein 1 | *FSCN1* | singed (Drosophila, homolog-like), actin bundling protein | Q16658 | organizes actin into parallel bundles; required for formation of stress fibers & cell protrusions | PMID: 21618240 |
| LIM domain proteins | actin cross-linking | LIM domain and actin binding 1 | *LIMA1* | EPLIN | Q9UHB6 | inhibits actin filament depolymerization & crosslinks filaments in bundles; required for cytokinesis | PMID: 26350886, 32496561 |
| leucine zipper protein | actin cross-linking | leucine zipper protein 1 | *LUZP1* | fimbacin, LUZP | Q86V48 | regulates actin-associated proteins (including ACTR2) at centrosome; interacts with LIMA1 & FLNA; LUZP1 depletion promotes ciliogenesis | PMID: 30990684, 32496561, 32553112 |
| gelsolin / villins | actin severing & capping | gelsolin | *GSN* | - | P06396 | regulates assembly & disassembly of actin filaments; binds (+) ends, preventing monomer addition; severs filaments and promotes nucleation to produce new filaments | PMID: 28814713, (23749648) |
| ENAH/VASPs | actin elongation & bundling | ENAH actin regulator | *ENAH* | mammalian enabled (MENA) | Q8N8S7 | induces the formation of F-actin rich outgrowths; implicated in many processes dependent on cytoskeleton remodeling & cell polarity | PMID: 30601697 |
| non-muscle myosin heavy chains | acto-myosin contraction | myosin heavy chain 9 & 10 | *MYH9, MYH10* | NMMHCA, NMMHCB | P35580, P35579 | complex with light chains to form non-muscle myosin II; regulates the actin cytoskeleton through acto-myosin contraction and stress fiber formation; mediates cell adhesion, cellular reshaping, migration & division | PMID: 25881509, 25494100 |
| myosin light chains | acto-myosin contraction | myosin light chain 12A & B | *MYL12A, MYL12B* | MLCB, MRLC2 | P19105, O14950 | required to maintain the integrity of non-muscle myosin II | PMID: 21126233 |
| Rho-family GTPases | acto-myosin contraction & F-actin stabilization | Ras homolog family member A | *RHOA* | ARH12, ARHA, Rho12, RHOH12 | P61586 | mediates Rho signaling during assembly of focal adhesions & actin stress fibres; active (GTP-bound) form activates effector proteins, including ROCKs, that regulate cytoskeletal dynamics, cell migration and the cell cycle | PMID: 17488776 |
| serine/threonine protein kinase | actin cytoskeleton regulation | Rho associated coiled-coil containing protein kinase 1 & 2 | *ROCK1, ROCK2* | p160ROCK, p164ROCK | Q13464, O75116 | activated by RhoA and phosphorylates downstream effectors (LIMK1, LIMK2, MYL9, FHOD1); regulates actin cytoskeleton organization, stress fiber and focal adhesion formation | PMID: (31904392) |
| zinc finger protein | actin cytoskeleton regulation | spalt-like transcription factor 1 | *SALL1* | ZNF794, TBS | Q9NSC2 | mutated in Townes-Brocks syndrome (TBS) causing aberrant degradation of LUZP1 & deregulating ciliogenesis | PMID: 29395072, 32553112 |
| retinal proteins | trafficking of actin modulators | photoreceptor cilium actin regulator | *PCARE* | C2orf71, RP54 | A6NGG8 | retinal specialist protein; facilitates actin remodeling (through WASF3) during formation of photoreceptor outer segment discs | PMID: 32312818 |
| beta thymosins | actin monomer capping & sequestration | thymosin beta 4 X-linked | *TMSB4X* | TMSB4 | P62328 | binds & sequesters G-actin monomers; inhibits actin polymerization | PMID: (29321224) |
| profilins | actin sequestration & monomer nucleotide exchange | profilin 1 | *PFN1* | - | P07737 | binds & sequesters G-actin monomers, catalyzing the exchange of ADP for ATP; low levels enhances polymerization whereas high levels inhibits polymerization | PMID: **32470361**, (32661903, 31811707) |
| tropomyosins | actin binding | tropomyosin 1 | *TPM1* | C15orf13, CMH3 | P09493 | binds & stabilizes actin filaments in muscle and non-muscle cells | PMID: 31054005 |
| coronins | actin binding | coronin 1A | *CORO1A* | Clabp, TACO | P31146 | required for the formation of membrane protrusions | PMID: 26916159 |
| cortactin | actin binding | cortactin | *CTTN* | EMS1 | Q14247 | activates & recruits ARP2/3 to actin filaments to promote branching; functions in Rho-GTPase signaling, vesicular trafficking & acto-myosin contraction | PMID: 29162307 |
| actin-depolymerizing factor homology | actin binding | drebin-like protein | *DBNL* | SH3P7, actin binding protein 1 (ABP1) | Q9UJU6 | neuronal F-actin binding proteins required for formation of cellular protrusions | PMID: 9693358 |
| cytoplasmic dynein | ciliary resorption | dynein light chain Tctex-type 1 | *DYNLT1* | t-complex-associated-testis-expressed 1-like 1(TCTEL1) | P63172 | controls ciliary resorption through regulation of branched actin polymerization and endocytosis | PMID: 28607034, 21394082 |
| serine/threonine protein kinase | ciliary disassembly | Aurora A kinase | *AURKA* | BTAK, AurA, STK7, ARK1 | O14965 | cell cycle-regulated kinase required for ciliary disassembly prior to mitosis; phosphorylates & activates PLK1; also mediates microtubule formation & stabilization | PMID: (26869233) |
|  |  | polo-like kinase 1 | *PLK1* | PLK | P53350 | required for ciliary disassembly prior to mitosis; phosphorylated & activated by AURKA; activates HDAC6 | PMID: 23345402, (26869233) |
| histone deacetylases | ciliary disassembly | histone deacetylase 6 | *HDAC6* | protein phosphatase 1, regulatory subunit 90 (PPP1R90) | Q9UBN7 | deacetylates axonemal tubulin; mediates ciliary disassembly with AURKA | PMID: (26651415), **24667272, 30044986** |
| WRAD / ATAC complex | basal body migration | WD repeat domain 5 | *WDR5* | SWD3, CFAP89 | P61964 | cilia and flagella-associated regulator of F-actin in multiciliated cells; binds basal bodies & mediates apical migration | PMID: **30205038** |
| phosphate (P) loop ATPase | basal body migration & docking | nucleotide binding protein 1 | *NUBP1* | NBP35, CIAO5 | P53384 | required for basal body migration, spacing and docking in multi-ciliated cells | PMID: 23685253 |
| ezrin/radixin/moesin (ERM) proteins | basal body docking | ezrin | *EZR* | villin 2 (VIL2) | P15311 | membrane-cytoskeleton linker protein that can bind F-actin; required for basal body docking in multi-ciliated cells | PMID: 25516973 |
| focal adhesion proteins | basal body migration & docking | protein tyrosine kinase 2 | *PTK2* | focal adhesion protein (FAK) | Q05397 | required for basal body migration, docking and spacing in multi-ciliated cells, formation & disassembly of focal adhesions & cell protrusions; reorganizes actin cytoskeleton and regulates cell migration & adhesion | PMID: 27895123, (24434137) |
| centrosomal proteins | protein scaffolding | pericentrin | *PCNT* | PCNT2, kendrin (KEN) | O95613 | component of pericentriolar material; required for mitotic spindle & centrosome formation, and microtubule organization | PMID: 30054381 |
|  | centriolar cap | centriolar coiled-coil protein 110 | *CCP110* | CEP110, CP110 | O43303 | inhibits ciliogenesis, with CEP97, by capping distal end of the mother centriole during maturation; required for anchoring of basal bodies to cell membrane | PMID: 17719545, 26965371 |
|  |  | centrosomal protein 97 | *CEP97* | leucine-rich repeats and IQ motif containing 2 (LRRIQ2) | Q8IW35 | inhibits ciliogenesis, with CCP110, by capping distal end of the mother centriole during maturation; required for recruitment of CCP110 to the centrosome | PMID: 17719545, 30404837 |
| nephrocystin proteins | ciliary protein | nephrocystin 3 | *NPHP3* | nephronophthisis 3, Meckel syndrome 7 (MKS7) | Q7Z494 | inhibits Disheveled-1-induced canonical Wnt signaling activity and may regulate non-canonical Wnt signaling & planar cell polarity | PMID: 18371931 |
| intraflagellar transport proteins | ciliary protein | clusterin associated protein 1 | *CLUAP1* | flagella associated protein 22 (FAP22), cilia and flagella associated protein 22 (CFAP22), intraflagella protein 38 (IFT38) | Q96AJ1 | mediates anterograde transport of ciliary proteins; may modulate the actin cytoskeleton | PMID: 29615496 |
|  |  | intraflagellar transport 88 | *IFT88* | tetratricopeptide repeat domain 10 (TTC10), polaris homologue | Q13099 | mediates anterograde transport of ciliary cargos; essential for ciliogenesis and implicated in autophagy, mediating the trafficking of ATG16L | PMID: 21441926 |

**Supplementary Table 1: Actin-binding and ciliary proteins.** Details of the protein families, illustrated by exemplar actin-binding and ciliary proteins, mentioned in this article. Key references are indicated by PMIDs: publications in brackets indicate reviews and bold text indicates studies that provide primary date to support a role in ciliogenesis or other ciliary process.
